# Supplementary material for: Splice-Junction-Based Mapping of Alternative Isoforms in the Human Proteome
Source: Cell Rep. Author manuscript; Available in PMC 2020 Jan 15. (PMC6961840; doi:10.1016/j.celrep.2019.11.026)
Supplement: 2 [file NIHMS1546469-supplement-2.zip › DF1/PXD010154/Esophagus/Esophagus_22_PPFIBP1_KAAALEQMDGIIAGSK.pdf]

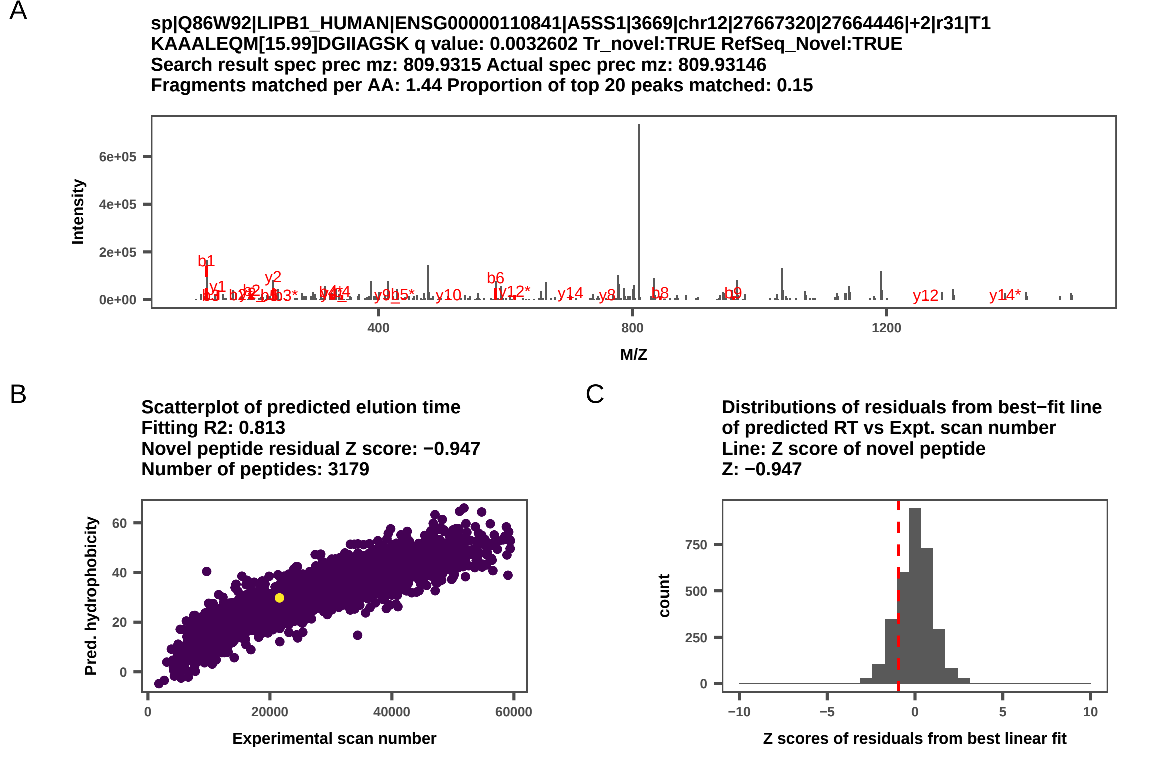
A
sp|Q86W92|LIPB1_HUMAN|ENSG00000110841|A5SS1|3669|chr12|27667320|27664446|+2|r31|T1
KAAALEQM[15.99]DGIIAGSK q value: 0.0032602 Tr_novel:TRUE RefSeq_Novel:TRUE
Search result spec prec mz: 809.9315 Actual spec prec mz: 809.93146
Fragments matched per AA: 1.44 Proportion of top 20 peaks matched: 0.15
Intensity
6e+05
4e+05
2e+05
0e+00
b1
y1
y2
b6
y12*
y10
y14
y8
b8
b9
y12
y14*
400
800
1200
M/Z
B
Scatterplot of predicted elution time
Fitting R2: 0.813
Novel peptide residual Z score: −0.947
Number of peptides: 3179
Pred. hydrophobicity
60
40
20
0
20000
40000
60000
Experimental scan number
C
Distributions of residuals from best−fit line
of predicted RT vs Expt. scan number
Line: Z score of novel peptide
Z: −0.947
count
750
500
250
0
−10
−5
0
5
10
Z scores of residuals from best linear fit
